# Safety and accuracy assessment of static computer assisted localized piezoelectric alveolar decortication: an in vitro study

**DOI:** 10.1007/s00784-024-05920-y

**Published:** 2024-12-02

**Authors:** María Lara-Muros, Octavi Camps-Font, Javi Vilarrasa, Jordi Vilarrasa, Javier Mir-Mari, Rui Figueiredo, Eduard Valmaseda-Castellón

**Affiliations:** 1 Oral Surgery and Implantology, Faculty of Medicine and Health Sciences, University of Barcelona, Barcelona, Spain; 2 Dental and Maxillofacial Pathology and Therapeutics Research Group, IDIBELL Research Institute, Barcelona, Spain; 3 Department of Periodontology, International University of Catalonia, Barcelona, Spain; 4 Orthodontics and Dentofacial Malformations, Faculty of Medicine and Health Sciences, University of Barcelona, Barcelona, Spain; 5 Facultat de Medicina i Ciències de la Salut, Campus de Bellvitge Universitat de Barcelona C/ Feixa Llarga s/n, Pavelló de Govern; 2a planta, Despatx 2.9, L’Hospitalet de Llobregat, 08907 Spain

**Keywords:** Accelerated tooth movement, Orthodontic treatment, Corticotomy, Piezocision, Computer-guided surgery, Accuracy

## Abstract

**Objectives:**

To assess the safety and accuracy of static computer assisted corticotomy surgery (sCACS) in comparison with freehand piezocision.

**Materials and methods:**

A randomized in vitro study was conducted. A total of 260 interradicular corticotomies were performed in 20 identical printed models. sCACS was performed in half of the models, while the rest underwent freehand localized decortication. Accuracy was measured in the three spatial axes by overlapping the digital planning with a previous cone-beam computed tomography (CBCT) scan of the patient and a postoperative CBCT of the models. Safety was determined as the number of damaged root surfaces. Descriptive and bivariate analyses were performed.

**Results:**

Freehand corticotomies increased the likelihood of iatrogenic root damage 2.21-fold (95%CI: 1.30 to 3.77; *p* = 0.004). Both groups showed some degree of deviation compared to digital planning. Nevertheless, the accuracy of sCACS was significantly greater in sagittal (B = -0.21 mm, 95%CI: -0.29 to -0.12; *p* < 0.001), axial (B = -0.32 mm, 95%CI: -0.48 to -0.18; *p* < 0.001) and angular deviation (B = -2.02º; 95%CI: -2.37 to -1.66; *p* < 0.001) compared to freehand surgery, with the exception of depth.

**Conclusions:**

The precision and safety of sCACS are greater than the freehand technique.

**Clinical relevance:**

Corticotomies are performed in crowded areas where there is usually space limitation. Clinicians should consider the systematic use of surgical guides, since minimal deviations can cause iatrogenic root damage in areas where malocclusions are present.

## Introduction

Over the last few decades, orthodontic treatments have undergone considerable development, affording faster and more esthetical outcomes. Several techniques have been introduced in order to accelerate orthodontic tooth movement, reduce adverse events and/or increase dental arch stability [1]. Among all these techniques, Corticotomy Accelerated Orthodontics (CAO) has been described as an effective surgical approach to accelerate orthodontic tooth movement [2]. A corticotomy is an intentional cut in the cortical bone, in contrast to osteotomy, which includes the cancellous bone as well [3, 4].

The underlying biological basis of the increase in tooth movement is found in the Regional Acceleratory Phenomenon (RAP) [5]. In this sense, any bone injury induces a transient demineralization-remineralization process and a dramatic increase in bone turnover, which results in transient osteopenia. Thus, the bone becomes less dense, although preserving its volume.

The first corticotomy attempts involved extensive decortications and full-thickness flaps, which increased the surgical time and patient morbidity [6]. Corticotomies have evolved to overcome these drawbacks. For instance, performing flapless procedures [7], using piezoelectric knives instead of surgical burs [8], or adding simultaneous grafting if needed [9, 10]. Recently, augmented corticotomy-assisted orthodontics have been associated to a wide range of tooth movements, less trauma to the periodontium, and modification of the gingival phenotype [11, 12]. More long-term studies are needed to confirm these results, however.

Piezocision™ is a flapless procedure popularized by Dibart et al. [13, 14]. The 3 mm deep corticotomy is performed though vertical microincisions on the buccal side of the alveolar bone. Hard and soft tissue grafting can eventually be performed via selective tunneling. According to Charavet et al. [15–17], Piezocision™ reduces the overall treatment time by more than 50% without increasing the risk of adverse events. However, some studies have reported root resorption or iatrogenic root damage (IRD) [18, 19], as interradicular corticotomies might impinge on the roots - especially when severe crowding is present.

An innovative approach with three-dimensionally printed Computer-Aided Design/ Computer-Aided Manufacturing (CAD/CAM) surgical guides has been introduced [20–27]. In this sense, static computer assisted corticotomy surgery (sCACS) not only allows safer and more accurate procedures but also reduces patient discomfort [21, 25]. However, to the best of the authors’ knowledge, no study has been specifically conducted to evaluate the safety and precision of these devices compared to the conventional freehand technique.

Hence, the aim of this randomized in vitro study was to compare the safety, in terms of iatrogenic root damage, between the sCACS system and the conventional freehand Piezocision™ technique. Additionally, the study sought to assess the deviations in 3D directions between the virtually planned and the actual interradicular piezoelectric corticotomies for both approaches.

## Materials and methods

A randomized in vitro study was carried out to evaluate the safety and accuracy of sCACS versus the freehand localized piezoelectric alveolar decortication technique. The study protocol complied with the Declaration of Helsinki guidelines and was approved by the Clinical Research Ethics Committee of the Dental Clinic of the University of Barcelona (Barcelona, Spain) (Protocol ref.: 30/2018).

**Fig. 1:**
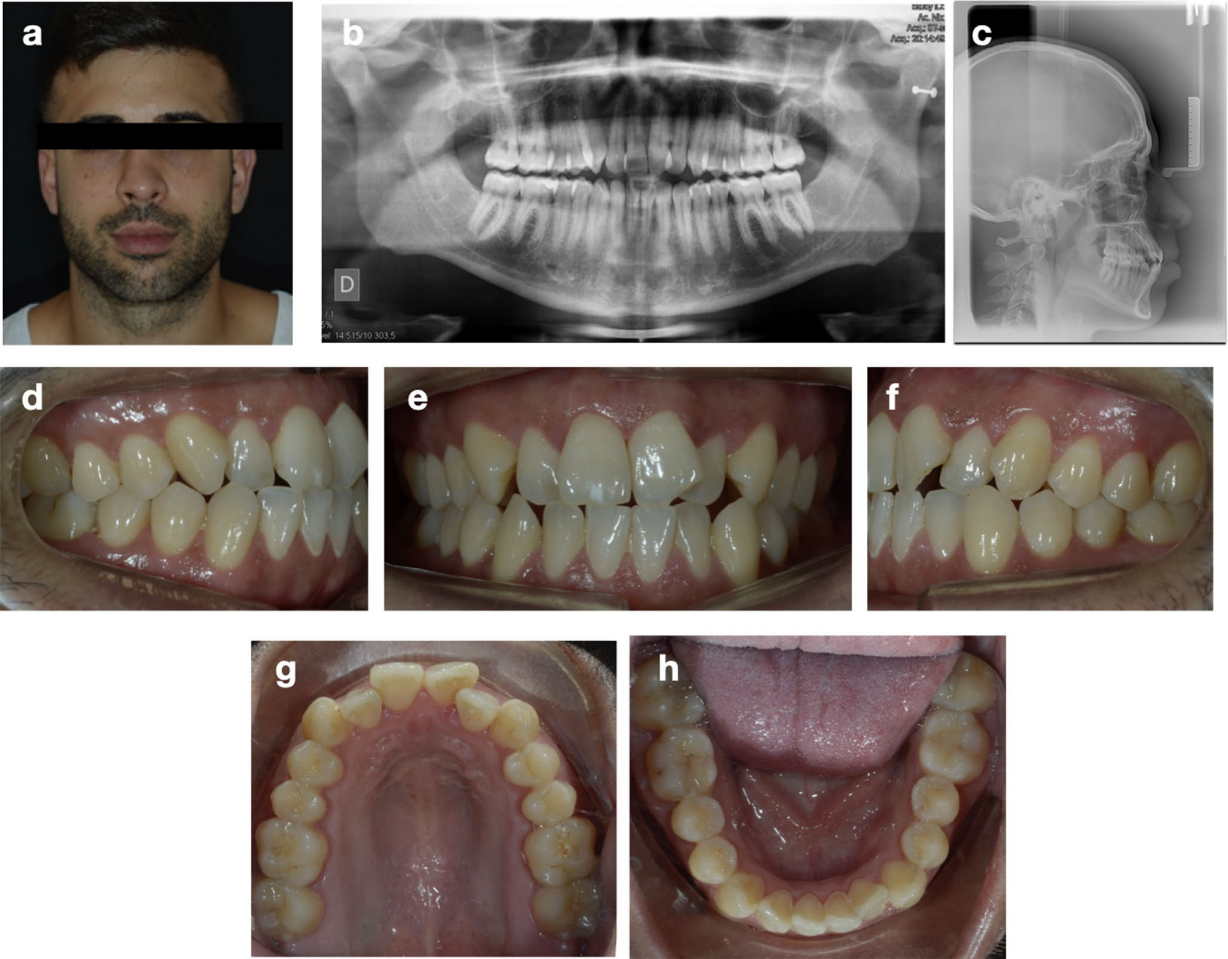
Case presentation. Pretreatment facial photograph **(a)**, panoramic **(b)** and lateral cephalometric radiographs **(c)**, and intraoral photographs **(d-h)**

A healthy 30-year-old male requiring orthodontic treatment in both dental arches was selected for this study for model manufacture (Fig. 1). The case was considered an appropriate candidate and representative to Piezocision™ because it involved full dentition excluding third molars, periodontal health and the absence of gingival recessions [15]. Ricketts cephalometric analysis revealed a Class I skeletal relationship (convexity 0.5 mm) with maxillomandibular dentoalveolar protrusion. Intraoral and dental cast examinations showed bilateral Class I molar and canine malocclusion with severe anterior crowding (11 mm in the maxilla and 7 mm in the mandible).

### Sample size

Sample size was calculated using the software G*Power v.3.1.3 (Heinrich- Heine Universität, Dusseldorf, Germany) based the assumption that a decrease in the risk of IRD of at least four times would be clinically significant. Considering an 8% risk of injury in sCACS established from pilot testing [28], an allocation ratio of 1:1, a risk of 0.05, and a statistical power of 80%, a total of 43 independent interradicular corticotomies per group were seen to be needed. Since the cuts were not independent due to the two-level data structure (model and cut), the number of models needed to be corrected. Assuming an intrasubject correlation of 0.35 (moderate) and that 13 interradicular corticotomies were planned on each model, 128 cuts (10 specimens) per group were required.

### Data acquisition and planning

**Fig. 2:**
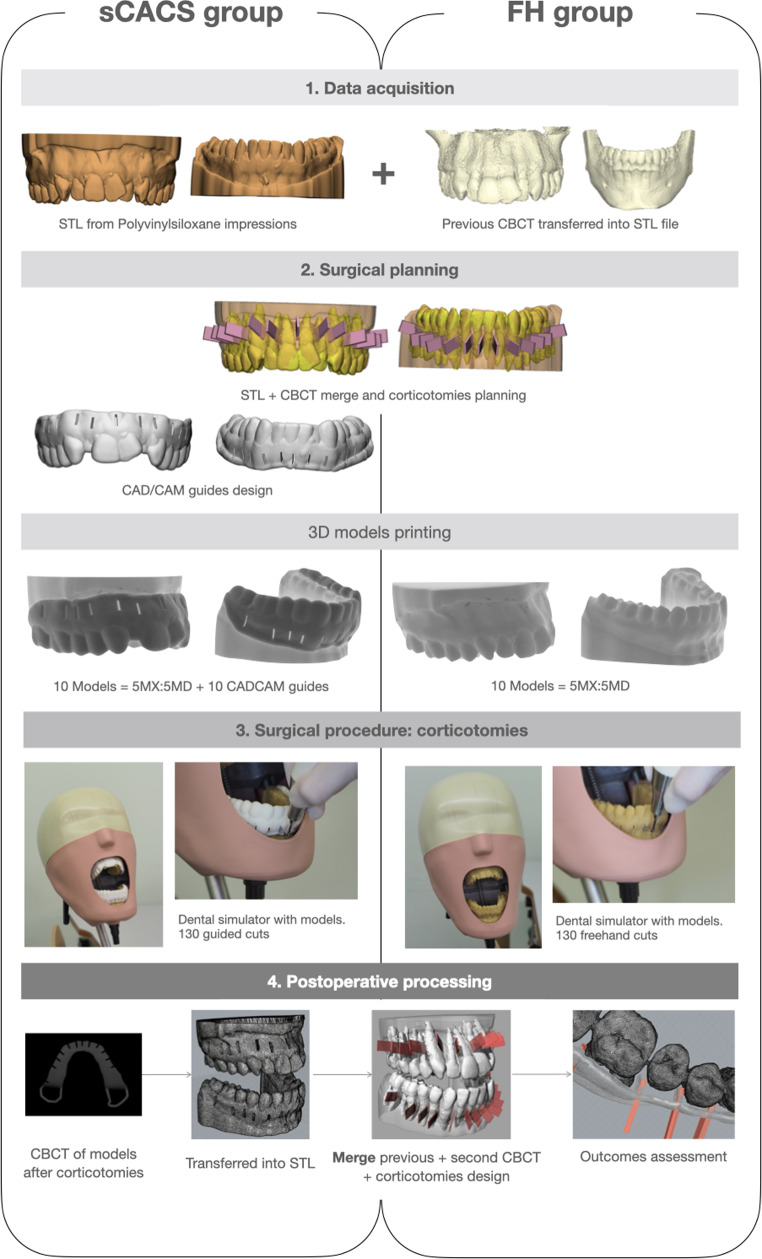
Workflow in each study group. sCACS: Static computer-assisted corticotomy surgery; FH: Freehand surgery; STL: Standard Triangulation Language; CBCT: Cone-beam computed tomography; CAD/CAM: Computer-Aided Design / Computer-Aided Manufacturing. Mx: Maxilla; Md: Mandible

Polyvinylsiloxane impressions of both arches of the patient were taken carefully up to the fornix (Aquasil Light body^®^ and Aquasil Soft Putty^®^Dentsply Sirona, York, PA, USA), following the one-step technique and poured in stone (Ortoguix - Protechno, Girona, Spain). Plaster casts were digitalized as Standard Tessellation Language (STL) files using a 3Shape TRIOS^®^ 3D scanner (3Shape A/S^®^ Copenhagen, Denmark) (Fig. 2).

The Digital Imaging and Communication in Medicine (DICOM) files obtained from the cone-beam computed tomography (CBCT) (Planmeca ProMax^®^ 3D Mid; Planmeca, Helsinki, Finland) scan of the patient were overlapped with the STL of the casts. Using 3D planning software (Exocad DentalCAD; exocad GmbH, Darmstadt, Germany), an individualized digital set-up was created. Interradicular corticotomies were virtually planned based on the protocol described by Dibart et al. [14] from mesial of the right second molar to mesial of the left second molar of both arches at sites with at least 1 mm of interdental bone. Accordingly, a surgical guide with piezosurgery slots was manufactured.

Twenty identical digital models (10 for each jaw) were printed using a digital light processing 3D printer (ProJet^®^ MPF 2500 Plus; 3DSystem; CA, USA), containing a light curing urethane acrylate resin (VisiJet^®^ M2R-TN, 3DSystem; CA, USA). Once printed, the models were dried and fully polymerized using an LC-3DPrint Box (3DSystem; CA, USA). Ten surgical guides with 2 mm thickness and 0.03 mm guide-to-teeth offset were printed with Formiga P110^®^ (EOS, Munich, Germany).

### Surgical procedure

All procedures were performed by the same clinician (M.L-M), a third-year post-graduate student in oral surgery with previous experience in Piezocision™. Before each instrumentation, models were screwed to a preclinical learning dental simulator reproducing a real clinical scenario. Guide templates were randomly assigned to 10 of the models (5 upper and 5 lower). Corticotomies were made with a PZ1 blade (Piezocision™; ActeonGroup, Merignac, France) in D1 mode, mounted on a Piezotome Solo™ ultrasonic device (Satelec^®^; ActeonGroup, Merignac, France) under copious irrigation. The surgical protocol followed the method described by Dibart et al. [14]. Accordingly, each model underwent 13 interradicular cuts, with each cut being 5 mm long and 3 mm deep. In the sCACS group, corticotomies were guided by the slots, and depth was verified using a periodontal probe (CP 15; Hu-Friedy, Chicago, IL, USA). In the freehand group, the depth was determined by referencing the 3 mm mark on the tip (Fig. 2).

### Postoperative processing

Immediately after cuts were made, a CBCT scan of each model was obtained (Planmeca ProMax^®^ 3D Mid (Planmeca, Helsinki, Finland) with 90Kv, 10 mA, 13.9 s, 1245 DAP (mGy*cm^2^), 0.4 mm voxel size) and exported as a STL file. The virtual surgical planning was aligned with the study specimens using the Orient3pt command in Rhinoceros 3D^®^ software (Robert McNeel & Associates^®^, Seattle, WA, USA), with the interincisal midline and the mesiobuccal cusps of the first molars serving as fiducial markers. This alignment was performed with a discrepancy of 1000 μm and a tolerance range of ± 100 μm. Finally, the resulting DraWinG file was imported into AutoCAD^®^ software (Autodesk^®^, San Rafael, CA, USA) for the assessment of outcome variables (Fig. 2).

### Outcome variables

The main outcome variable was the risk of IRD, defined as the occurrence of corticotomy impinging on a root, as determined through visual inspection from a 3D view of the render.

**Fig. 3:**
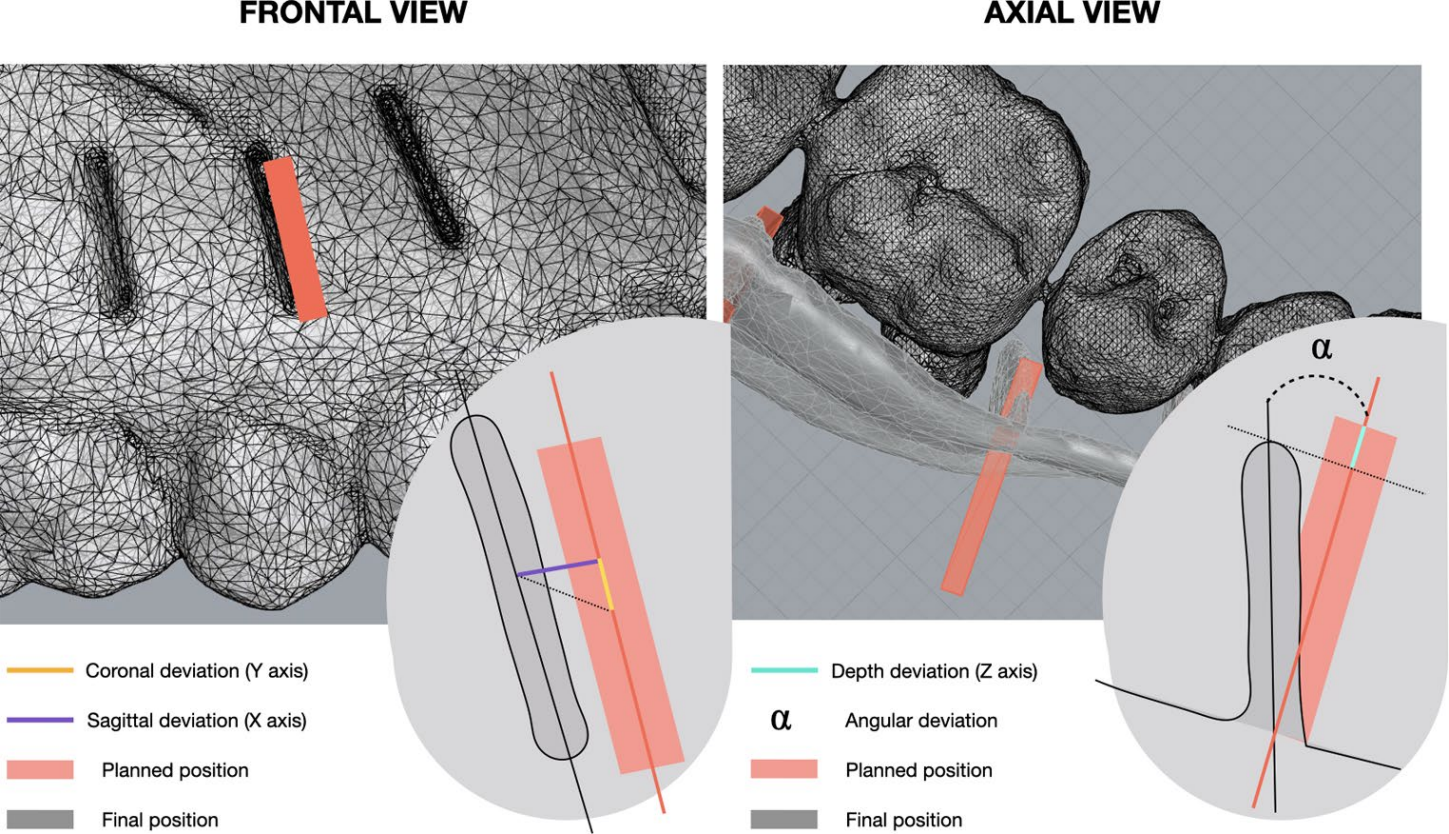
Deviation outcomes. Measurement deviation between the planned position (in red) and the final position (in grey)

The secondary outcome variables were (Fig. 3):

- Sagittal deviation: defined as the horizontal or mesio-distal distance between the planned and the final corticotomy along the X-axis, expressed in mm.
- Axial deviation: vertical or corono-apical distance between the virtually planned and the actual interradicular piezoelectric corticotomy along the Y-axis, expressed in mm.
- Coronal deviation: depth deviation along the Z-axis by comparing the digital plan with the actual corticotomy performed, expressed in mm.
- Angular deviation: defined as the largest angle between the longitudinal axes of the planned and final corticotomy, measured in decimal degrees (°).

To avoid observation bias, safety and accuracy assessments were conducted by a single trained researcher (O.C.-F.) who was blinded to the technique employed. To test intraexaminer reliability for IRD, an assessment of 3 randomly selected models (90 measurements) was repeated after 2 weeks.

### Statistical analysis

Categorical outcomes were presented as absolute and relative frequencies. The normality of scale variables was explored using the Shapiro-Wilk test and by visual analysis of the P-P plot and box plot. Where normality was rejected, the median and interquartile range (IQR) were calculated. Where data distribution was compatible with normality, the mean and standard deviation (SD) were used.

Multilevel binary logistic and linear regression models were conducted to evaluate safety and accuracy outcomes based on the guidance method using generalized estimating equations (GEE), respectively. The GEE method was used to account for the fact that repeated observations (several corticotomies) were available for a single model. Group (sCACS or freehand), location (maxilla or mandible) and region (anterior or posterior) were entered as predictor variables. The interaction effects between the exposure variable (i.e., group) for both location and region were also explored. Adjusted beta coefficients for regression models including 95% confidence intervals (95%CIs) were obtained from the Wald χ^2^ statistic.

All analyses were conducted using the SPSS version 27 statistical package (IBM Corp, Armonk, NY, USA). Statistical significance was considered for *p* < 0.05.

## Results

A total of 260 corticotomies (130 with sCACS and 130 freehand) were assessed without registering any protocol deviation.

### Iatrogenic Root damage

**Fig. 4:**
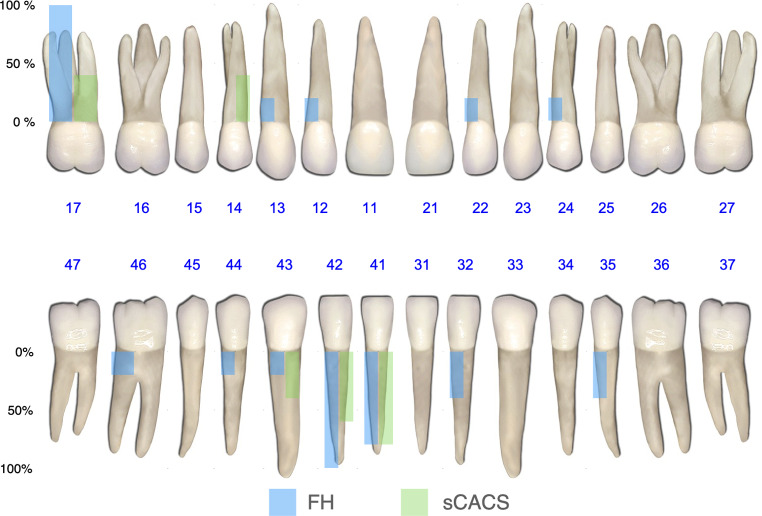
Iatrogenic root damage location and relative frequency. sCACS: Static computer-assisted corticotomy surgery; FH: Freehand surgery

Twenty-five piezoelectric corticotomies in the freehand surgery (FH) group (17.14%, 95%CI: 12.49 to 23.06) and 12 in the sCACS group (8.56%, 95%CI: 5.90 to 12.25) caused IRD (ORa = 0.45, 95%CI: 0.27 to 0.77; *p* = 0.004). In both groups, IRD were more likely in the mandible (ORa = 2.21, 95%CI: 1.34 to 3.65; *p* = 0.047) and in anterior areas (ORa = 2.44, 95%CI: 1.01 to 5.90; *p* = 0.047) (Fig. 4). The Cohen’s kappa coefficient (κ) was 1, thus indicating perfect agreement.

### Accuracy outcomes

**Fig. 5:**
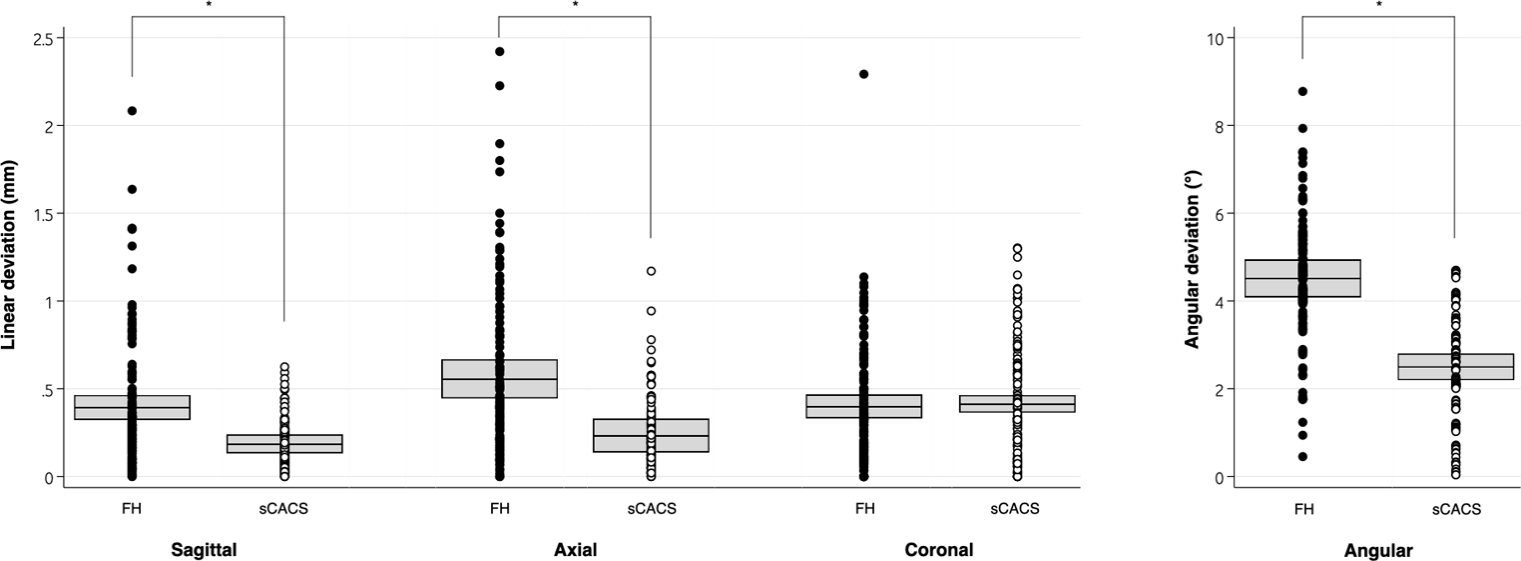
Boxplot illustrating the deviation analysis in both groups and in the three spatial axes. sCACS: Static computer-assisted corticotomy surgery; FH: Freehand surgery. *Statistically significant difference

Accuracy analyses revealed that sCACS significantly reduced the sagittal (B = -0.21 mm, 95%CI: -0.29 to -0.12; *p* < 0.001), axial (B = -0.32 mm, 95%CI: -0.48 to -0.18; *p* < 0.001) and angular deviations (B = -2.02º; 95%CI: -2.37 to -1.66; *p* < 0.001) (Table 1; Fig. 5).

None of the interactions yielded statistically significant differences for any of the accuracy variables assessed (*p* > 0.05), i.e., the differences in IRD and deviations between groups did not depend on the position of the corticotomy.

**Table 1:** Summary of accuracy variables. sCACS: static computer-assisted corticotomy surgery; FH: Freehand surgery; SD: standard deviation; MD: Mean difference (sCACS – FH); 95%CI: 95% confidence interval. Note: MD adjusted according to the generalized estimating equations (GEE), considering other covariables. *Statistically significant difference

Accuracy variable	sCACS Mean (SD)	FH Mean (SD)	MD (95%CI)	*P*-value
Sagittal (mm)	0.19 (0.14)	0.39 (0.37)	-0.21 (-0.29 to -1.24)	< 0.001^*^
Axial (mm)	0.23 (0.20)	0.56 (0.47)	-0.32 (-0.46 to -0.18)	< 0.001^*^
Coronal (mm)	0.41 (0.33)	0.40 (0.34)	0.01 (-0.06 to 0.09)	0.715
Angular (º)	2.50 (1.27)	4.51 (1.35)	-2.02 (-2.37 to -1.66)	< 0.001^*^

## Discussion

To the best of the authors’ knowledge, this study represents the first attempt to specifically investigate, using an in vitro experimental design, whether the use of a static guided surgery system enhances the safety and accuracy of Piezocision™ surgery. In this regard, according to the results obtained, sCACS significantly improves both the safety and accuracy of the procedure.

Although localized piezoelectric alveolar decortication can be applied in various orthodontic scenarios, it has traditionally been recommended for addressing crowding in adult patients, thus reducing treatment time and modifying the periodontal phenotype [29]. For this reason, the selected case (i.e., class I molar and canine malocclusion with severe anterior crowding) was chosen to be representative of this condition. However, it is important to note that in cases of crowding, the risk of damage may be higher due to root proximity. In this regard, Patterson et al. [18] noted that nearly one third of the patients had considerable IRD from the piezocision surgical procedure. In order to reduce this risk and minimize trauma, several static guided surgery approaches have been proposed [21, 24, 27]. In the present investigation, IRD was reported in 8.56% of the guided corticotomies (95%CI: 5.90 to 12.25). This figure was much higher in the control group, where the incidence of IRD could reach 23%. Accordingly, the sCACS technique was associated with a 2.21-fold decrease in the likelihood of IRD (95%CI: 1.30 to 3.77; *p* = 0.004). Notably, in both the sCACS group and the control group, most cases of IRD occurred in the mandible and in the anterior region, which is usually the most crowded area (Fig. 4). Consequently, given that evidence suggests the RAP effect after corticotomy extends approximately 1 cm beyond the cut, it may be advisable to avoid areas with extreme root proximity (i.e., < 1.5 mm) and to select strategic incision sites [21, 30, 31]. Nonetheless, additional long-term randomized controlled clinical trials are necessary to fully elucidate the clinical relevance of these findings.

Cassetta and Ivani [21] previously assessed the accuracy of computer-guided Piezocision™ surgery at the entry point and depth deviation in a clinical prospective pilot study. They reported a mean deviation of 0.67 mm (range: 0.0 to 1.44; SD = 0.31) at the entry point and 0.54 mm (range: 0.17 to 0.80; SD = 0.21) in depth, which were higher than those observed in the present study (Table 1). In contrast, the results of a recent randomized controlled trial associated sCACS with deviations from the digital plan of only 0.05 mm (range: 0.0 to 0.10; SD = 0.03), 0.08 mm (range: 0.0 to 0.15; SD = 0.06), and 0.15 mm (range: 0.0 to 0.20; SD = 0.10) on the X, Y, and Z axes, respectively [25]. These discrepancies could mainly be attributed to differences in study design, the location where the corticotomies were performed, and the design of the surgical guide.

Regarding angular deviation, although no prior evidence was found for either freehand or computer-assisted Piezocision™, our findings are in line with those previously stated in dental implant studies [32–35]. Particularly, in the sCACS group, the angular deviation was less than 4º (Mean = 2.50º; SD = 1.27), which was nearly half of that observed in controls (Mean = 4.51º; SD = 1.35).

Our results indicate that sCACS was associated with significantly less deviation compared to freehand cuts for all variables, except for depth. A possible explanation for this might be the opacity of the guide and the method used for intraoperative depth assessment. In this sense, while in the freehand technique the knife landmark was used as a depth gauge, in guided decortications a periodontal probe was employed, thus potentially introducing some discrepancy. To minimize this error and improve visibility of the depth, a translucent template could be used, as suggested by Hou et al. [23]. They also recommended employing rigid materials with multiple pores to provide better support during guidance and allow greater access for saline irrigation to the surgical site [23]. Other authors advocate for the use of slots to ensure highly controlled alveolar decortications [21]. Moreover, although no apparent signs of damage or deformation were observed in the surgical guides due to the action of the piezoelectric device, attention should be paid to the design and composition of the guide slots, as these factors can influence its precision and durability [21, 36]. Given these considerations, a comparative study is needed to determine the most effective and safest design.

Even though the present research could not analyze the role of experience, current evidence from implant dentistry suggest that computer assisted systems may be particularly beneficial for novice clinicians. Specifically, these investigations found that when these methods are used, both experienced and novice professionals achieved similar levels of accuracy [37–39]. In this context, the fact that the corticotomies were performed by a third-year postgraduate student may have influenced the results, potentially leading to an overestimation of the reported effect sizes. Therefore, future studies should be conducted to assess the impact of the operator’s experience on the outcomes of localized piezoelectric alveolar decortication procedures.

In this study, all models were replicas of a single case. This homogeneity limits the study’s ability to account for variability in anatomical structures and patient-specific factors, which could affect the external validity and applicability of the findings to a broader population. Interestingly, upon analyzing the operator’s performance over time, significant improvements were observed in IRD (*p* = 0.006), as well as in sagittal (*p* < 0.001) and axial (*p* = 0.043) deviations in the sCACS group. Conversely, no improvements were found in the conventional freehand method. This suggests that, as in other areas of dentistry, the learning curve is much steeper when using computer-assisted systems [39].

Although sCACS has demonstrated high accuracy and safety in localized piezoelectric alveolar decortication, it is not without drawbacks. These include complex fabrication processes, time consumption, and higher costs compared to the freehand technique [29, 40]. Additionally, clinicians cannot adapt their surgical planning during surgery, the obstructed water flow to the tip of the piezoknife might lead to overheating, and the surgeon’s perception can be affected by the presence of a splint [29]. To overcome some of these limitations, the use of dynamic CACS has been proposed. Currently, there is only a case report evaluating dynamic CACS in Piezocision™ with promising results [41]. Nevertheless, further research is needed to corroborate these findings and fully establish the benefits and limitations of this technology in this specific application.

This study has some limitations that need to be addressed. First, in vitro studies have limited generalizability, as they often do not replicate the complexities of clinical settings, such as patient malocclusion, the degree of mouth opening, and other intraoral conditions that can influence the outcomes of interventions [29]. Furthermore, the materials used may not accurately replicate the mechanical properties and behavior of natural tissues, potentially affecting the observed outcomes. This limitation could particularly lead to an overestimation of the IRD risk in the control group, as root palpation —a useful tool in freehand techniques—could not be simulated in the models. Blinding was another challenge in the study, as the surgeon was aware of the treatment being performed due to the specific requirements of the sCACS technique. To minimize the impact of this issue, the researcher responsible for overlaying the preoperative and postoperative STL files and collecting the outcome data was blinded to the technique used. Finally, although the intra-examiner reliability of the evaluator was perfect, the fact that the measurements were taken manually using the virtually planned decortication may have led to a subjective interpretation. Therefore, future research on this topic should implement automatic postoperative processing methods to minimize these errors [42].

## Conclusions

Static computer assisted corticotomies reduce the risk of root damage and increase the accuracy of piezocision surgery. Clinicians should be aware that, even when surgical guides are used, iatrogenic root damaged may occur.

## Data Availability

The data presented in this study are available on request from the corresponding author (Prof. Dr. Octavi Camps Font).
